# *Lacticaseibacillus* Strains Isolated from Raw Milk: Screening Strategy for Their Qualification as Adjunct Culture in Cheesemaking

**DOI:** 10.3390/foods12213949

**Published:** 2023-10-29

**Authors:** Luca Bettera, Alessia Levante, Elena Bancalari, Benedetta Bottari, Martina Cirlini, Erasmo Neviani, Monica Gatti

**Affiliations:** Department of Food and Drug, University of Parma, 43124 Parma, Italy; luca.bettera@unipr.it (L.B.); elena.bancalari@unipr.it (E.B.); benedetta.bottari@unipr.it (B.B.); martina.cirlini@unipr.it (M.C.); erasmo.neviani@unipr.it (E.N.); monica.gatti@unipr.it (M.G.)

**Keywords:** raw milk cheese, cheese ripening, NSLAB, adjunct culture, fermented food, cheese flavor

## Abstract

The microbial ecology fundamentals of raw milk and long-ripened cheeses consist of a complex interaction between starter lactic acid bacteria (SLAB) and non-starter LAB (NSLAB). Although NSLAB aromatic properties are paramount, other phenotypic traits need to be considered for their use as adjunct cultures, such as the capability to endure technological parameters encountered during cheesemaking. The present study focused on the isolation and characterization of NSLAB from spontaneously fermented raw cow’s milk coming from 20 dairies that produce Grana Padano PDO cheese. From 122 isolates, the screening process selected the 10 most diverse strains belonging to *Lacticaseibacillus* spp. to be phenotypically characterized. The strains were tested for their growth performance in milk in combination with the application of technological stresses, for their ability to produce volatile compounds after their growth in milk, and for their ability to use different nutrient sources and resist chemicals. The complex characterization qualified the strains 5959_Lbparacasei and 5296_Lbparacasei as the best candidates to be used as adjunct strains in the production of raw milk and long-ripened cheeses, provided that antibiotic resistance is measured before their employment. Other strains with interesting aromatic capabilities but lower heat resistance were 5293_Lbparacasei, 5649_Lbparacasei and 5780_Lbparacasei, which could be candidates as adjunct strains for uncooked cheese production.

## 1. Introduction

Microbiological and biochemical changes in the curd are crucial factors in producing raw milk hard cheeses characterized by long ripening. Different European cheeses fall into this category, some examples are the Italian Parmigiano Reggiano PDO (Protected Designation of Origin), Grana Padano PDO (the PDO cheese most exported in the world), Asiago PDO, Nostrano Valtrompia PDO, the Swiss Gruyère PDO and Sbrintz PDO, the French Beaufort PDO and the Austrian Vorarlberger Bergkäase PDO. Manufacturing of traditional cheeses of this category relies on a defined set of technological operations, as well as a complex microbiota that plays a key role in defining the quality of the final cheese product.

The microbial ecology distinguishing this cheese variety is largely influenced by the technological processes that are applied during cheesemaking, so much so that two different microbial populations develop successively: firstly, the starter lactic acid bacteria (SLAB), deliberately added mainly for curd acidification, followed by non-starter LAB (NSLAB), adventitious milk contaminants correlated to cheese flavor formation during ripening [1,2,3].

The NSLAB moiety of raw milk hard cheeses is mainly composed of facultatively heterofermentative lactobacilli, belonging to the *Lacticaseibacillus* genus, which was recently updated regarding its taxonomy [4]. This taxonomic clade includes the species *Lacticaseibacillus casei*, *Lacticaseibacillus paracasei* and *Lacticaseibacillus rhamnosus*, which were previously defined as the *Lb. casei* group, due to their phylogenetic relatedness [5].

These strains enter the cheesemaking process from raw cow’s milk, where they are not the prevalent genus [6] but possess adaptation capabilities that allow them to survive the initial steps of the manufacturing process, making *Lb. casei* group the dominant microbiota of raw milk hard cheeses during the subsequent ripening process [7,8]. Indeed, during the early raw milk hard cheese manufacturing, technological parameters (cooking temperature, pH decrease, salt exposure, eventual lysozyme addition) will ensure the quality of the produced cheese and prevent the development of spoilage and pathogen bacteria throughout the subsequent long ripening process [1,2].

Therefore, NSLABs need to possess strategies to adapt to the varying environment of the ripening cheese, and strains belonging to the *Lb. casei* group could benefit from the ability to ferment different carbohydrates [9] allowing them to develop in the cheese matrix after SLAB has depleted lactose. Another aspect that could improve their fitness in the cheese environment is the capability of entering a dormancy state [10], where environmental conditions do not hamper microbial viability, turning into a metabolically active and dividing state when the stress factors are released, after the brining step, as shown by various studies that focused on the dynamics of raw milk hard cheeses microbiota during ripening [11,12,13].

The development of NSLAB in long-ripened cheeses is associated with the formation of the peculiar organoleptic properties of the final product and for this reason, strains belonging to the *Lb. casei* group has been proposed as adjunctive cultures for the dairy industry [14]. Indeed, several studies have proposed the characterization of NSLAB belonging to the *Lb. casei* group of cheese origin, aiming to characterize the capability of these strains to produce volatile compounds in milk [15] or in cheese-mimicking substrates [16], for the informed selection of strains to be used as an aromatic adjunct starter for cheesemaking.

Another approach is to focus on *Lb. casei* isolates that originate from raw cow’s milk used for cheese manufacturing, as reported by Bancalari and colleagues, who used impedance analysis to measure isolates’ technological performance and studied the volatile profile to identify potential adjunct strains for the dairy industry [17]. The raw milk was also used as a source for isolating potential probiotic strains [18,19,20] or starters LAB [21]. The advantage of isolating bacteria from raw milk rather than cheese may be the higher choice of species and strains since the raw milk arbors a higher biodiversity in comparison to the ripened cheese [22].

The purpose of this work was to isolate from raw milk NSLAB that could be used as adjunct culture in cheesemaking. Raw cow’s milk was sampled from 20 dairies that produce Grana Padano PDO cheese located in northern Italy.

The isolates were phenotypically characterized, but although the aromatic properties of the selected strain(s) are paramount, other phenotypic traits need to be considered, such as the capability to endure the technological parameters encountered during cheesemaking. In this perspective, the strains have been first genotypically characterized, followed by a screening of their metabolic activity and chemical sensitivity by phenotypic microarray. The impedance technique was used to evaluate the strains’ growth performance in milk in combination with the application of thermal stress, salt, and lysozyme. Finally, the production of volatile compounds after their growth in milk was assessed, using the headspace solid-phase microextraction (HS-SPME) technique coupled with gas chromatography–mass spectrometry (GC-MS).

This multifactorial approach allows us to highlight the genotypic and phenotypic biodiversity of strains from the raw milk microbial community while collecting data on their different suitability for dairy applications by evaluating adaptation and aromatic properties.

## 2. Materials and Methods

### 2.1. Raw Milk Sampling

The raw milk intended for processing into Grana Padano PDO cheese was sampled from 20 dairies located in 12 provinces of northern Italy. More precisely, vat raw milk (pH = 6.71 ± 0.03; temperature = 16.07 ± 6.46 °C), consisting of a mix of partially skimmed milk by overnight spontaneous creaming and morning whole milk in a 50:50 *v*/*v* ratio, was delivered to the laboratory in refrigerated conditions.

### 2.2. Strain Isolation and Screening

#### 2.2.1. Raw Milk Spontaneous Fermentation

Raw milk samples (100 mL) were immediately incubated anaerobically at 8 °C for 60 days in order to perform a spontaneous fermentation and select the subdominant NSALB strains, as previously proposed [17]. At the end of the incubating period, a simple odor evaluation was performed by the operators to assess the intensity of the following aroma descriptors on a scale from 0 (absent) to 5 (strong): expired milk (off-flavor), acidic (yogurt-like) and cheesy.

#### 2.2.2. Bacteria Culturing and Isolation

The spontaneous fermented raw milk was analyzed for the lactobacilli viable count; furthermore, the pH was measured using a pH meter Beckman Instrument mod F350 (Fullerton, CA, USA) and glass electrode (Hamilton, Bonaduz, Switzerland)

Samples (1 mL) were tenfold serially diluted in sterile Ringer’s solution (Oxoid, Ltd., Basingstoke, UK), and plated (dilution ranging from 10^−2^ to 10^−7^) on acidified de Man, Rogosa and Sharpe agar [23] (MRSa; pH = 5.4 reached by acetic acid addition; Oxoid). Plates were incubated at 37 °C for 48 h in anaerobic conditions with a gas pack (Fisher Scientific, Rodano, Italy) in MRS broth. The lactobacilli concentration was calculated as colony-forming units per mL (CFU/mL).

A number between 5–7 colonies for each sample were selected maximizing their macroscopic diversity (i.e., size, thickness, shape, color) using a Nikon Eclipse Si microscope (Nikon Europe, Firenze, Italy). The number was further reduced by screening the most morphologically different isolates based on descriptors of cell length (short, medium, long) and organization (single rod, diplo–strepto-bacilli). Isolates were purified by streaking repeatedly on MRS agar, then grown in MRS broth and stored at −80 °C with the addition of 30% *v*/*v* of glycerol (solution 50% *v*/*v* glycerol-distilled H_2_O).

Three strains belonging to the University of Parma Culture Collection (UPCC) were used in this study as a reference, i.e., strains *Lb. rhamnosus* 1473, *Lb. casei* 2094 and *Lb. paracasei* 4186, isolated from ripened cheeses and characterized at the genome level [10].

#### 2.2.3. DNA Extraction and Strain Identification

Bacterial genomic DNA was extracted using the DNeasy Kit (Qiagen, Milan, Italy) following the manufacturer’s instructions.

The strains belonging to the *Lb. casei* group were identified by doing an endpoint species-specific PCR targeting the *spxB* gene. The primers used were *poxcDNAFw* 5′-CAGACGCAATGATCAAGGTG-3′ and *poxPromRv* 5′-AATGCGCC**Y**ACTTCTTCATG-3′ as proposed by [13]. Each PCR mixture contained 1 μL of each primer at a concentration (10 μM), 10 μL of 2X GoTaq Master Mix (Promega, Madison, WI, USA), 7 μL of sterile MilliQ water and 1 μL of DNA (final volume, 20 μL). The following amplification conditions were used: initial strand denaturation at 95 °C for 10 min, followed by 30 cycles of 95 °C for 30 s, 58 °C for 30 s and 72 °C for 1 min, and a final extension step at 72 °C for 7 min. The PCR results were checked with a 1% agarose electrophoresis gel run. Discrimination among the *Lb. casei*/*paracasei* and *Lb. rhamnosus* species of the isolates was performed according to the melting temperature curve analysis method, a post-PCR method described by Levante et al. (2017) [13]; however, all the newly isolated strains belonged to the *Lb. paracasei* species, as confirmed by corresponding melting peak temperatures (Figure S1).

The strains that did not belong to the *Lacticaseibacillus* genus were identified by the amplification and sequencing of the 16S rRNA gene. The primers used were 46Fw 5′-GC**Y**TAACACATGCAAGTCGA-3′ and 536Rv 5′-GTATTACCGCGGCTGCTGG-3′ [24]. Each PCR mixture contained 1 μL of each primer at concentration [10 μM], 10 μL of 2X GoTaq Master Mix (Promega, Madison, WI, USA), 7 μL of sterile Milli-Q water, and 1 μL of DNA (final volume, 20 μL). PCR products were checked on 1.5% *w*/*v* agarose gel in 1 × TAE buffer at 90 V/cm for 20 min and visualized on a GelDoc Go gel imaging system (BioRad, Milan, Italy). The amplified sequences were compared with reference sequences using the Basic Local Alignment Search Tool (BLAST^®^, https://blast.ncbi.nlm.nih.gov/Blast.cgi, accessed on 1 February 2022) against the 16S ribosomal RNA sequences database.

#### 2.2.4. Strain Genotypic Characterization

The isolates belonging to the *Lacticaseibacillus* genus were further screened to select the most genotypically different. The Amplified Fragment Length Polymorphisms (AFLP) technique was applied following the protocol improved by [25].

Briefly, after a restriction–ligation step, the digested-ligated DNA underwent a pre-amplification using the pre-selective primers *Eco*RI-0 5′-GACTGCGTACCAATTC and *Mse*I-0 5′-GATGAGTCCTGAGTAA; finally, a selective PCR using the primers *Eco*RI-A 5′-GACTGCGTACCAATTCA and *Mse*I-A 5′-GATGAGTCCTGAGTAAA was carried out. The selective primers were previously checked for their efficiency using the tool in silico AFLP-PCR amplification [26].

First, a restriction–ligation (R/L) step was performed, and the reactions were composed of 10 μL of 250 ng/μL BSA (New England Biolabs, Ipswich, MA, USA), 5 μL of 1× T4 DNA ligase buffer with 1 μL of 1 mM ATP (New England Biolabs, Ipswich, MA, USA), 1 μL of 50 pmol/μL MseI adaptor, 1 μL of 5 pmol/μL EcoRI adaptor, 1 μL of 10 mM ATP (Invitrogen S.R.L., Milano, Italy), 0.5 μL of 10 U/μL MseI (New England Biolabs, Ipswich, MA, USA), 0.25 μL of 20 U/μL EcoRI (New England Biolabs, Ipswich, MA, USA), 0.1 μL of 200 U/μL of T4 DNA ligase (New England Biolabs, Ipswich, MA, USA) and 15 μL of template DNA (500 ng), and volume was adjusted to 50 µL with Milli-Q water. The reaction was incubated at 37 °C for 4 h. After the R/L step, the digested-ligated DNA underwent two amplification reactions that were executed after incubation. The first pre-selective PCR included 10 μL GoTaq^®^ Master Mix 2× (Promega, Madison, WI, USA), 0.6 μL of 10 μM EcoRI-0 Primer (5′-GACTGCGTACCAATTC-3′), 0.6 μL of 10 μM MseI-0 Primer (5′-GATGAGTCCTGAGTAA-3′) and 2 μL of digested-ligated DNA, until a final volume of 20 μL. Amplified samples were diluted 1:10 in TE (10 mM TRIS HCl at pH = 8, 0.1 mM EDTA at pH = 8). The selective amplification reaction was composed of 10 μL GoTaq^®^ Master Mix 2× (Promega, Madison, WI, USA), 0.6 μL of 10 μM EcoRI-A Primer (5′-GACTGCGTACCAATTCA-3’), 0.6 μL of 10 μM MseI-A Primer (5′-GATGAGTCCTGAGTAAA-3′) and 2 μL of diluted DNA, until a final volume of 20 μL. All PCRs were performed in a GeneAmp^®^ PCR System 2700 (Applied Biosystem, Foster City, CA, USA) by following the thermocycler program of Bertani et al. (2019) [25]. Amplifications were followed by electrophoresis on a 1.2% agarose gel in 1 × TAE buffer at 90 V/cm for 20 min and visualized as previously described for 16S gene PCR products, to confirm successful PCR reactions. AFLP profiles were compared through Bionumerics version 8.1 (Applied Maths NV, Sint-Martens-Latem, Belgium) by setting GeneScan-500 (LIZ) as the size standard to normalize peaks. Bands with a length ranging between 50 and 500 bp were considered. AFLP profiles were compared by the Jaccard similarity coefficient and clustered according to the Unweighted Pair Group Method with the Arithmetic Mean (UPGMA) method. The correlation similarity cut-off was set at 40% according to the similarity coefficient obtained for biologically independent replicates of type strains used as reference, i.e., *Lb. rhamnosus* 1473, *Lb. casei* 2094 and *Lb. paracasei* 4186.

### 2.3. Strain Phenotypic Characterization

#### 2.3.1. Evaluation of the Strain Resistance to Cheesemaking Stresses

**Figure 1:**
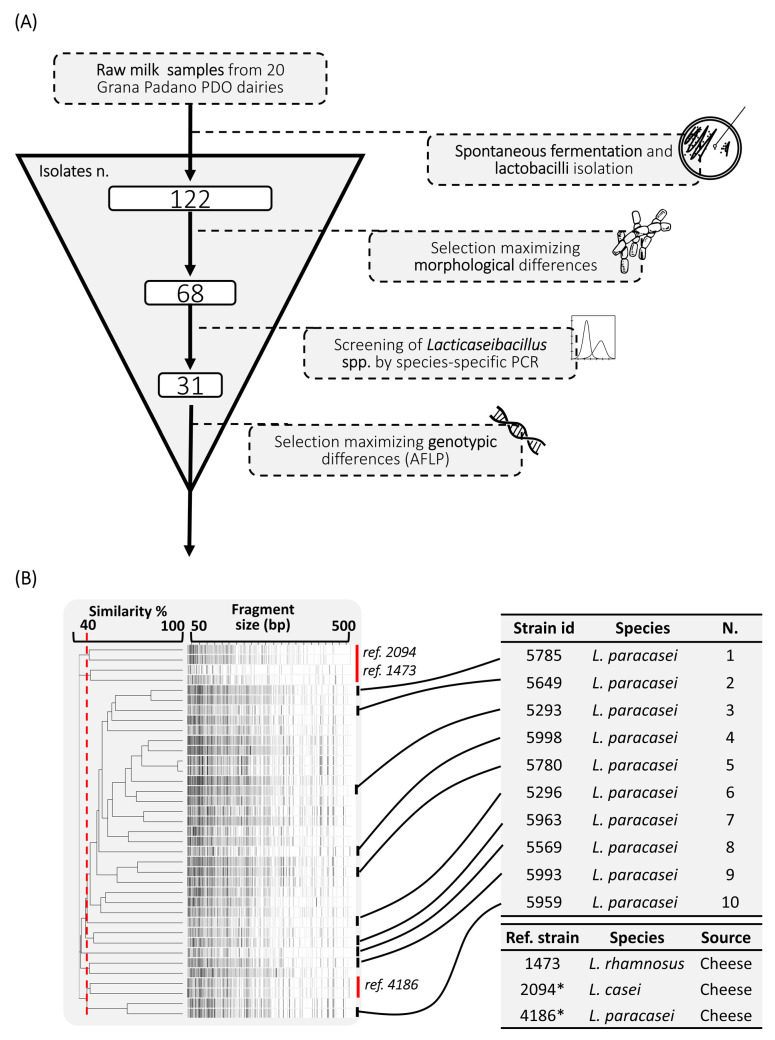
Strains screening schematization: (**A**) isolation and selection of 31 *Lacticaseibacillus* spp.; (**B**) selection of the 10 most genotypically diverse strains based on their clusterization using the UPGMA (Unweighted Pair Group Method with Arithmetic Mean) method on AFLP (Amplified Fragment Length Polymorphisms) profiles’ Jaccard similarity co-efficient; a similarity cut-off set of 40% was used to create the clusters. * = reference strains selected as controls for the phenotypic study.

The 12 strains selected from the screening (10 isolates and 2 references, Figure 1) were evaluated for their ability to grow in UHT partially skimmed milk (UPSM). Frozen broth cultures were revitalized by inoculation in 6 mL of MRS broth (3% *v*/*v*) and incubating overnight anaerobically at 37 °C. This propagation was repeated three times. The viable count of each strain after growing in MRS broth (t = 20 h) was calculated by plating on MRSa agar. Cultures were then propagated twice in UPSM (2% *v*/*v*) at the same conditions; viable counts on MRSa agar after growing in UPSM (t = 20 h) were also calculated (Table 1). Each culture was then tenfold diluted and inoculated in 6 mL of UPSM (2% *v*/*v*) in six different conditions: (i) UPSM; (ii) UPSM + NaCl (1% *w*/*v*); (iii) UPSM + NaCl (2.5% *w*/*v*); (iv) UPSM + NaCl (5% *w*/*v*); (v) UPSM + Lysozyme (200 mg/L) (Sigma Life Science, Merck KGaA, Darmstadt, Germany); and (vi) the culture was heat treated at 54 °C for 1 h and before the inoculation in UPSM. The inoculated sample was analyzed using impedometric analysis [27]. Briefly, a BacTrac 4300^®^ Microbiological Analyzer system (SY-LAB, Neupurkersdorf, Austria) was used to evaluate the impedometric curves. The M% values over time (48 h at 37 °C) were interpolated with the Gompertz model using DMFit v3.5 [28] (Baranyi & Roberts, 1994). The strain’s growth kinetic parameters extrapolated were Lag (h), adaptation time to the substrate before the growth; rate (−), acidification speed; and yEnd (M%), maximum acidification capacity. If the model was not applicable because of the absence of growth, the values used were Lag = 48 h, rate = 0, and yEnd = 0. The analysis was conducted in triplicate.

#### 2.3.2. Evaluation of Metabolic Capabilities

##### Carbon Source Utilization and Chemical Sensitivity

The Biolog GEN III MicroPlate (BIOLOG Inc.©, Hayward, CA, USA) was used to study the carbon sources utilization and chemical sensitivity of screened strains following the methodology proposed by Troiani and colleagues [29].

Briefly, for the preparation of the inoculum, pure cultures were grown on solid MRS agar media, and single colonies were picked from the plate and suspended in 12 mL of inoculation fluid IF-A (BIOLOG Inc.©, Hayward, CA, USA) until reaching the recommended cell density of 90–98% of transmittance (T) on the BIOLOG turbidimeter. The cell suspension was inoculated into the 96-well GEN III and incubated at the optimal strain’s temperature to allow phenotypic fingerprint. Metabolic activity and chemical sensitivity of strains were detected on the BIOLOG MicroReader Station (BIOLOG Inc.©, Hayward, CA, USA) by absorbance reading at a wavelength of 590 nm, before incubation and after 72 h. Measurement of the reduced tetrazolium violet dye (purple colored) indirectly represents the increased metabolic activity in wells where cells used the substrate [30].

The phenotypic raw data were elaborated by calculating the Average Well Color Development (AWCD) as reported in the literature [30,31]. To reduce the noise levels, all absorbance values of the carbon source utilization (GEN III microplate columns 1–9) were referenced against the negative control well (A1), while the absorbance values of the chemical sensitivity (GEN III microplate columns 10–12) were subtracted from half of the positive control well (A10), and subsequently all divided by the respective AWCD obtaining the Absorbance Ratios parameter (Table 2). Negative values were set to 0. Normalized data were used for statistical analysis in the R environment [32], using the UPGMA clustering method of the “stats” package. Results of metabolic and sensitivity assay were plotted in two different heatmaps using the “heatmaps” package [33].

##### Volatile Compounds

The 10 LAB strains were tested for their ability to produce volatile compounds in fermented milk following the method proposed by Bancalari et al. (2017) [17], with slight modifications. Briefly, cultures were revitalized and inoculated in 5 mL of UHT partially skimmed milk as reported in Section 2.3.1, and then incubated anaerobically for 4 days at 37 °C. The analysis was performed in duplicate. For each considered sample, the fermentation step was conducted directly in 20 mL glass vials.

The samples were then analyzed by Head Space Solid Phase Microextraction technique coupled with gas chromatography–mass spectrometry analyses (HS-SPME/GC-MS). Before analysis, 5 μL of Toluene (100 mg/L, Sigma, St. Louis, MO, USA) was added into the sealed vials to be used as an internal standard. Volatile compounds were extracted at 40 °C for 20 min, after an equilibration time of 20 min at the same temperature, and adsorbed on an SPME fiber functionalized with a divinylbenzene–carboxen–polydimethylsiloxane coating (Supelco Inc., Bellefonte, PA, USA).

All the analyses were conducted on a TRACE 1300 gas chromatograph (Thermo Fisher Scientific Inc., Milan, Italy) coupled to an ISQ mass spectrometer (Thermo Fisher Scientific Inc., Milan, Italy) equipped with an electronic impact source. Analytes were separated on a Supelcowax 10 capillary column (Supelco, Bellefonte, PA, USA; 30 m × 0.25 mm × 0.25 μm), applying a temperature ramp as follows: at starting oven temperature was set at 50 °C and maintained for 3 min, then it was increased at 130 °C (5 °C/min), and then at 220 °C (15 °C), and after that, the temperature was maintained at 220 °C for 10 min. The detection was performed by setting the mass spectrometer in full scan mode, registering the spectra in the mass range of 40–500 *m*/*z*.

Peak identification was performed both by comparison of the experimental mass spectra with those reported in the NIST14 instrument library and by the linear retention indexes (LRIs) calculation, based on the analysis of a C_8_–C_20_ alkane standard solution (Sigma-Aldrich, Milan, Italy) performed in the same instrumental conditions applied for sample characterization. A semi-quantitative approach was performed by comparison of the relative peak areas of the identified volatiles to the peak area of Toluene.

### 2.4. Statistical Analysis

To evaluate the significance of the factors “strain” and “analysis condition” in influencing the impedometric parameters during the resistance to cheesemaking stress test, the one-way ANOVA model was applied (significance level α = 0.05) using PROC GLM statement of SAS^®^ OnDemand for Academics (©2022 SAS Institute Inc., Cary, NC, USA). A post hoc LSMEANS test with *t* adjustment was applied to check the difference between the samples.

Impedometric and gas-chromatography results were subjected to principal component analysis (PCA) using the packages “FactoMineR” [34] and “factoextra” for the results plotting [35]. Furthermore, the Pearson coefficient for variables correlations was calculated and plotted, respectively, using the packages “stats” and “corrplot” [36]. Both the PCA and the Pearson correlation were conducted in the R environment.

## 3. Results

### 3.1. Screening of the Isolates

A schematization of the isolates’ screening strategy is reported in Figure 1. At the end of the incubation period (60 d at 8 °C anaerobically), all the raw milk samples developed a cheesy note, and in many of them (8 out of 20) this was perceived as a strong flavor. The acidic flavor was present in almost all the samples, while only two of them developed an unpleasant expired milk off-flavor. The spontaneous low-temperature fermented raw milks were rich in lactobacilli, having a viable count on an average of 7.23 log CFU/mL (range 8.55–5.76) and an average pH = 4.52 (range 4.69–4.31) (Table 3).

**Figure 2:**
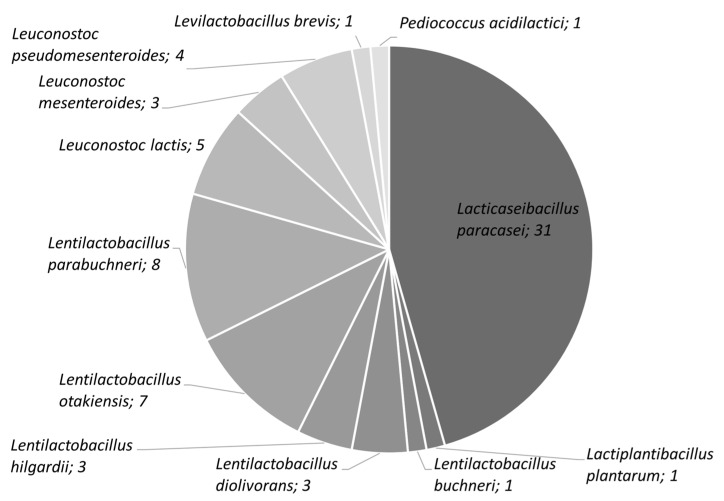
Strains isolated from spontaneous fermented raw milk and selected for species identification. *Lacticaseibacillus paracasei* were identified via species-specific PCR and AFLP clustering, with the remaining strains via 16S rRNA sequencing. The number of isolates is reported beside the species (tot. number = 68).

A total of 122 lactobacilli were isolated from all the samples. The 68 most morphologically different lactobacilli were selected for molecular identification (Figure 2). The species-specific PCR revealed that 31 of these isolates belonged to the *Lb. casei* group. Analyzing the melting temperature curves, the strains were discriminated as belonging to the *Lb. casei/paracasei* species (Figure S1). The remaining isolates belonged to the species *Lentilactobacillus parabuchneri* (*n* = 8), *Lentilactobacillus otakiensis* (*n* = 7), *Leuconostoc lactis* (*n* = 5), *Leuconostoc pseudomesenteroides* (*n* = 4), *Leuconostoc mesenteroides* (*n* = 3), *Lentilactobacillus hilgardii* (*n* = 3), *Lentilactobacillus diolivorans* (*n* = 3), *Pediococcus acidilactici* (*n* = 1), *Lentilactobacillus buchneri* (*n* = 1) and *Lactiplantibacillus plantarum* (*n* = 1).

The 31 strains belonging to the *Lb. casei* group, together with reference strains, were subjected to AFLP analysis. Samples were clustered with the UPGMA method on AFLP profiles’ Jaccard similarity coefficient (Figure 1B). Based on the 40% similarity cut-off set, the strains were grouped into 9 main clusters. All the isolates were clustered with a similar AFLP profile with the reference strain *Lacticaseibacillus paracasei* 4186 (4186_Lbparacasei). Ten strains with high genotypic diversity were selected for the phenotypic analysis, in addition to the reference strains *Lacticaseibacillus casei* 2094 (2094_Lbcasei) and 4186_Lbparacasei.

### 3.2. Phenotypic Tests

#### 3.2.1. Resistance to Cheesemaking Stresses

**Figure 3:**
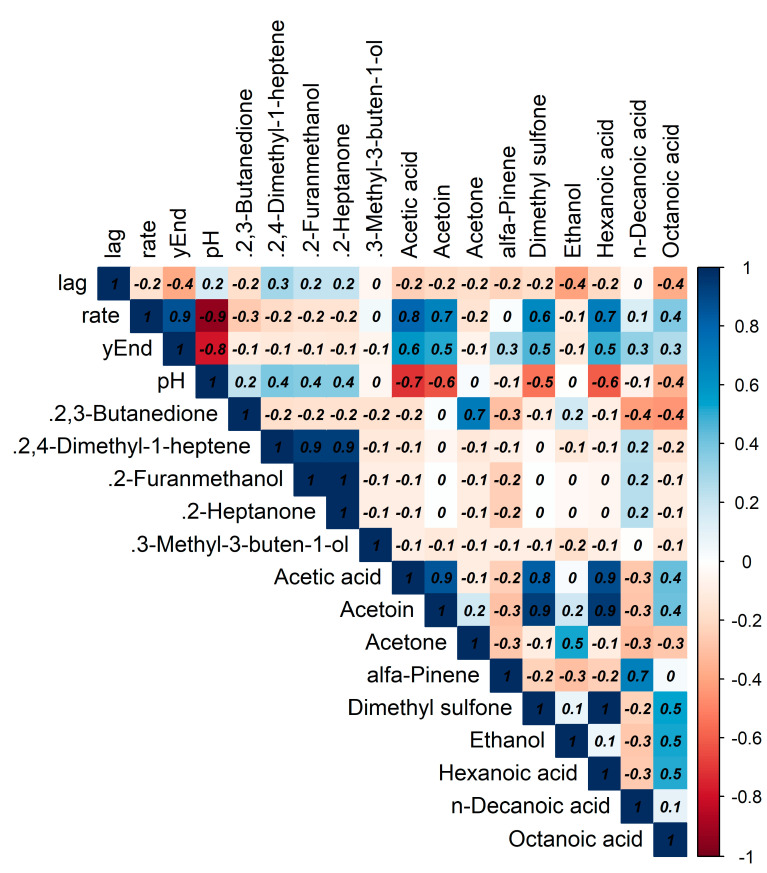
Pearson’s correlation between aromatic and impedometric features of the strains after their growth in milk.

**Figure 4:**
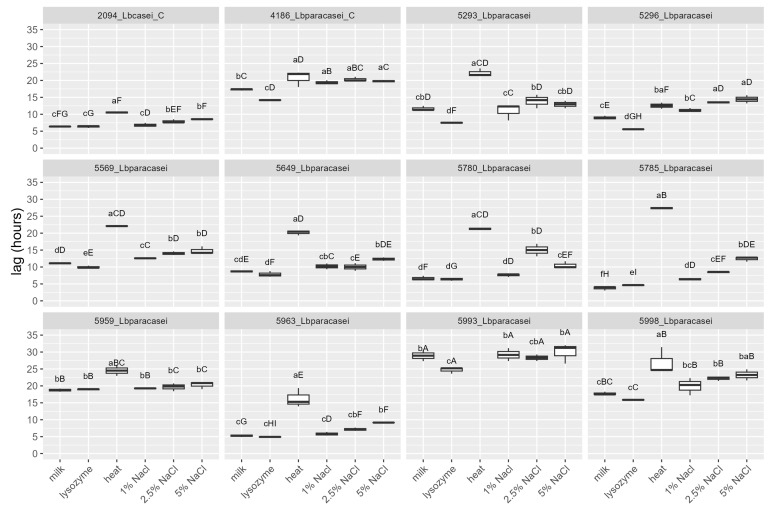
Lag values of the strains after their growth (48 h at 37 °C) in milk in different conditions: absence of stress (milk), in presence of 200 mg/L of lysozyme (lysozyme), after heat treatment of 54 °C for 1 h (heat) and in presence of different % (*w*/*v*) of NaCl. Growth conditions within the same strain with different lowercase letters are significantly different (*p* < 0.05); strains within the same growing condition with different uppercase letters are significantly different. _C = reference strains.

**Figure 5:**
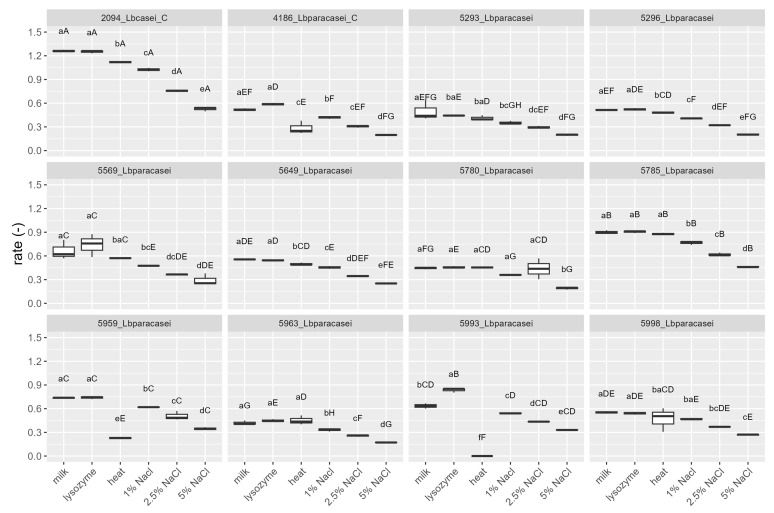
Rate values of the strains after their growth (48 h at 37 °C) in milk in different conditions: absence of stress (milk), in presence of 200 mg/L of lysozyme (lysozyme), after heat treatment of 54 °C for 1 h (heat) and in presence of different % (*w*/*v*) of NaCl. Growth conditions within the same strain with different lowercase letters are significantly different (*p* < 0.05); strains within the same growing condition with different uppercase letters are significantly different. _C = reference strains.

**Figure 6:**
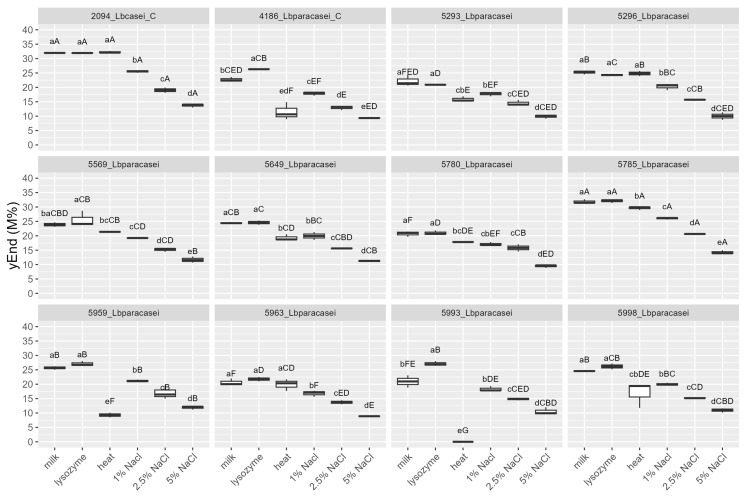
yEnd values of the strains after their growth (48 h at 37 °C) in milk in different conditions: absence of stress (milk), in presence of 200 mg/L of lysozyme (lysozyme), after heat treatment of 54 °C for 1 h (heat) and in presence of different % (*w*/*v*) of NaCl. Growth conditions within the same strain with different lowercase letters are significantly different (*p* < 0.05); strains within the same growing condition with different uppercase letters are significantly different. _C = reference strains.

**Figure 7:**
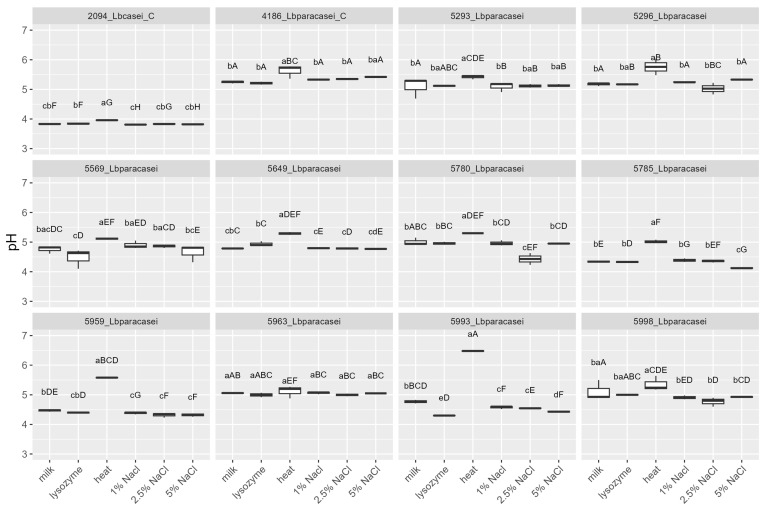
pH values of the milk after the strains’ growth (48 h at 37 °C) in different conditions: absence of stress (milk), in presence of 200 mg/L of lysozyme (lysozyme), after heat treatment of 54 °C for 1 h (heat) and in presence of different % (*w*/*v*) of NaCl. Growth conditions within the same strain with different lowercase letters are significantly different (*p* < 0.05); strains within the same growing condition with different uppercase letters are significantly different. _C = reference strains.

The strains were tested, by mean of impedometric analysis, for their ability to grow in milk in optimal conditions (no stress applied) and under the simulated main stresses encountered during raw milk hard cheesemaking, i.e., possible presence of lysozyme, heating at a curd cooking temperature ≈ 48–56 °C, and high NaCl concentration. Three parameters were used to describe the strains’ growth kinetic in response to the stresses: (i) Lag, measured in hours and representing the adaptation time of the strain before starting to grow, the higher the value, the greater the sensitivity to stress; (ii) rate, the slope of the kinetic curve indicating the acidification speed, the higher the value, the lower the sensitivity to stress; and (iii) yEnd, the maximum M% value measured and representing the strain’s acidification capacity, the higher the value, the lower the sensitivity to stress. Rate and yEnd are strongly positively correlated, and both are inversely correlated with the Lag (Figure 3). The results are reported in Figure 4, Figure 5 and Figure 6, together with the pH values reached in inoculated milk after the incubating period (Figure 7).

The strains 5785_Lbparacasei and 5963_Lbparacasei were the faster to adapt and start growing in milk in the absence of any stress, having Lag values, respectively, of 3.7 ± 0.6 h (mean ± SD) and 5.3 ± 0.4 h, which is significantly lower in comparison with the Lag parameter of other strains. The heating treatment was the stress that most affected the time needed for the strains to start growing, except for strains 4186_Lbparacasei (reference) and 5296_Lbparacasei which resisted this stress well. Then, 5993_Lbparacasei was not able to grow after the heating treatment; furthermore, it had a Lag parameter significantly higher in all the conditions tested. The influence of the stresses on the acidification speed and capacity was strain dependent. Excluding the reference strains, 5785_Lbparacasei was the isolate with the highest rate and yEnd, in all the growth conditions. In general, the rate and yEnd values were more affected by the salt addition, although strain 5959_Lbparacasei and 5993_Lbparacasei were sensitive also to the heating treatment. The pH values had a trend inversely correlated with rate and yEnd values, but they were less influenced by the different treatments among the strains.

#### 3.2.2. Aromatic Compounds Production

The results of the concentration of the volatile compounds produced by the strains after growing in milk are reported in Table 4. The calculated Kovats index and the reference for the compound identification are reported in Table S1. 5649_Lbparacasei was the isolate producing more ketones, in particular a high amount of Acetoin, and together with 5293_Lbparacasei and 5296_Lbparacasei, they were the only producing 2,3-Butanedione (diacetyl, who has sweet, creamy, buttery odor). The acid compounds were produced in higher concentrations by 5780_Lbparacasei and 5785_ Lbparacasei.

**Figure 8:**
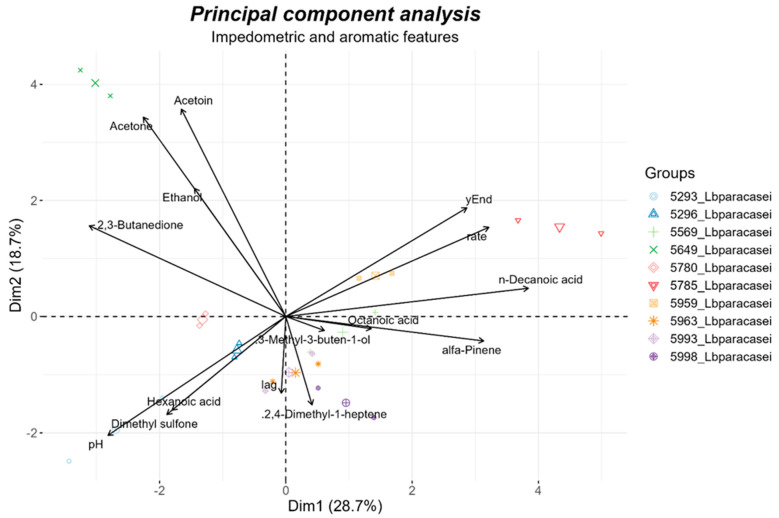
Principal component analysis of impedometric (*n* = 4) and aromatic (*n* = 10) features of strains after their growth in milk. Legend on the right reports symbols used for each sample replicate, a larger symbol inidicates the centroid between the replicates data.

The strains’ similarity based on their ability to produce aromatic compounds and growth in milk was assessed by reducing the dimensionality of both chemical (*n* = 11) and impedometric (*n* = 4) features by principal component analysis (PCA). The ordination biplot is shown in Figure 8; the features’ % contribution to the explained variance is reported in Figure S2. The most diverse strains were 5649_Lbparacasei, characterized by high acetone and 2,3-Butanedione production; 5785_Lbparacasei, which showed a good acidification capacity and produced a high amount of decanoic acid; and 5293_Lbparacasei, which showed low rate and yEnd but high dimethyl sulfone and hexanoic acid production. The strain 5959_Lbparacasei showed similar features to 5785_Lbparacasei, while all the other strains were less diverse among each other as demonstrated by their proximity in the biplot.

#### 3.2.3. Carbon Source and Sensitivity Assays

**Figure 9:**
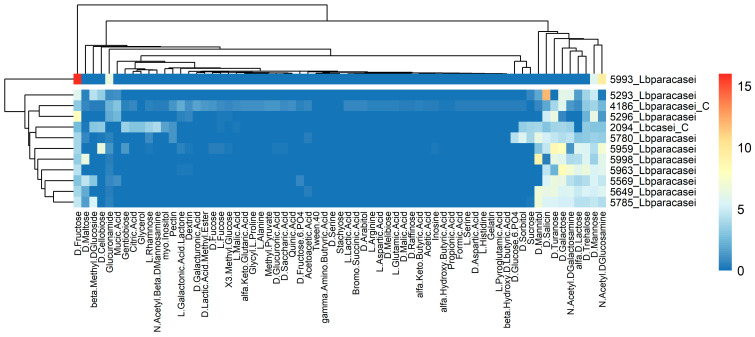
Strains clustering (vertical axis) based on their ability to grow on different carbon sources (horizontal axis). The growth rate increases from blue (no growth) to red.

**Figure 10:**
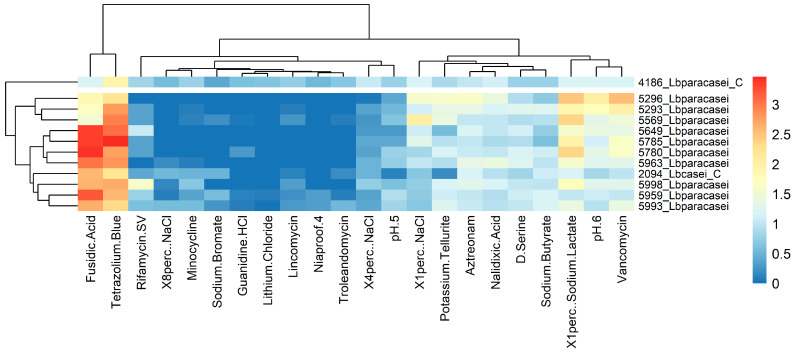
Strains clustering (vertical axis) based on their ability to resist different chemical inhibitors (horizontal axis). The growth rate increases from blue (no growth) to red.

The strains were analyzed for their ability to use different carbon sources as growth substrates, as well as their resistance to different chemicals. The results were used to cluster them based on their similar response and are reported separately for the two assays (Figure 9 and Figure 10). The strain 5993_Lbparacasei showed a singular profile, being able to grow very well using D-fructose and N-Acetyl-D-Glucosamine. The strain 5293_Lbparacasei was also very different mainly because of its ability to metabolize D-Salicin and D-Cellobiose. Not one of our isolates was able to grow using citric acid, while 5780_Lbparacasei, 5649_Lbparacasei and 5959_Lbparacasei were the only ones able to metabolize acetic acid. Regarding the chemical sensitivity, the strains 5296_Lbparacasei and 5293_Lbparacasei were particularly resistant to vancomycin, while 5649_Lbparacasei, 5785_Lbparacasei, 5780_Lbparacasei and 5959_Lbparacasei are resistant to fusidic acid.

## 4. Discussion

When produced with raw milk, the microbial ecosystem of traditional long-ripened hard cheeses harbors a non-starter microbiota that is correlated with a peculiar cheese flavor formation. The dominant bacteria of this ecosystem are the NSLAB, in particular the species belonging to the *Lacticaseibacillus casei* group, which contribute with their metabolic pathways to the cheese proteo-lipolysis as well as citrate degradation, so as to be proposed as adjunctive cultures for the cheese industry. In this study, we assessed some important phenotypic features (growth kinetics in milk; resistance to high temperature, salt and lysozyme; aroma production) of strains belonging to the *Lb. casei* group isolated from raw milk used for Grana Padano PDO cheese, to evaluate their potential use as adjunct strains in the production of raw milk, long-ripened hard cheeses.

### 4.1. Strains’ Resistance to Cheesemaking Stresses

The isolation and screening process allowed the selection of ten *Lb. paracasei* strains morphologically and genotypically diverse based on the microscope and AFLP profiles clustering evaluations. All the strains were able to grow in milk showing kinetic parameters comparable with those of other strains of the same species tested in the same conditions [15,17]. Strains 5785_Lbparacasei and 5963_Lbparacasei were characterized by a significantly shorter Lag, meaning earlier adaptation in milk. This feature could cause a possible competition in lactose metabolism with SLAB [3] during the early stages of the curd acidification process and compromise cheesemaking. However, exposure of the strains to typical stresses encountered during cheese manufacturing can affect kinetic parameters. Indeed, when the heat stress was applied (54 °C for 1 h), the response of the kinetic parameters varied in a strain-dependent fashion. For instance, 5993_Lbparacasei was not able to grow in the incubation condition tested, indicating a possible lower expression of heat-shock proteins (HSPs) and excluding this strain as adjunct in curd cooked cheese production. The inability of *Lb. paracasei* strains to grow at thermophilic temperatures (45 °C) was also reported by Fitzsimons et al. (1999) [37], who phenotypically characterized NSLAB isolated from mature Irish Cheddar cheeses. On the other hand, strains 5959_Lbparacasei, 5998_Lbparacasei and 5296_Lbparacasei showed good recovery to the heat stress and resistance to salt and lysozyme; furthermore, they did not show an excessively short Lag, but at the same time a good acidification capacity and rate, making them good candidates as adjunct strains.

### 4.2. Strains’ Metabolic Capabilities

Different studies have highlighted the genotypic diversity that is often found in isolates from the same niche, especially in the case of dairy products [38,39]. Genetic variability at the genus level is relevant in *Lacticaseibacillus*, as reported in various studies [4,5], and, to some extent, this variability has been recognized also during the different stages of raw milk hard cheese ripening [13]. However, there is a general awareness that similar phenotypes displayed by strains do not always correspond to similar or even closely related genotypes [40]. According to the results presented in this study, the genetic variability does not seem to be reflected in the capability of the strains to metabolize the different substrates of the Biolog GEN III plate. Most of the strains tested showed similar phenotypic profiles sharing the use of different carbon sources such as simple sugars (α-D-glucose, α-D-galactose, D-fructose, etc.) but also D-Gluconic acid and D-salicin (Figure 9). As expected, our strains were not able to ferment L-rhamnose since this distinguishes the species *Lb. casei/paracasei* from *Lb. rhamnosus* within the *Lb. casei* group [41] (Minervini & Calasso, 2022). The tested isolates were not able to use L-arginine as a growing substrate, indicating the possible absence of the arginine deaminase (ADI) pathway which is important for the adaptation in the cheese environment [42]. Regarding the synthesis of aromatic compounds, strains 5293_Lbparacasei, 5296_Lbparacasei and 5649_Lbparacasei were able to produce 2,3-butanedione (diacetyl), a ketone compound that is related to the pleasant sweet, creamy and buttery notes, and as a consequence of interest for the aroma formation of dairy products [43]. Furthermore, strains 5649_Lbparacasei and 5959_Lbparacasei produce the ketone compound acetoin, as well, which has pleasant buttery and creamy notes.

Phenotypic characterization of the selected strains included their sensitivity to various chemical compounds. The obtained results showed a widespread resistance to fusidic acid, an antibiotic increasingly used for the treatment of skin infections due to methicillin-resistant *S. aureus* [44]. On the other hand, the resistance to vancomycin, the last resort for the treatment of severe infections caused by Gram-positive bacteria such as *Enterococcus* species, *Staphylococcus aureus* and *Clostridium difficile* was limited [45]. Anyway, the use of GEN III was useful for a preliminary evaluation of these features, and this resistance should be evaluated and measured through more accurate and official antimicrobial susceptibility testing methods such as the agar diffusion method [46]. Usually, the evaluation of antibiotic resistance is required for the LAB to be used as a starter [47]. However, in the case of ripened cheeses, the vitality of this type of bacterial cells is destined to drastically decrease in a few months, contrary to the number of NSLAB cells which increase during cheese ripening and then decrease again after very long ripening times. This implies that NSLAB can be present and viable in large amounts when the cheese is consumed, for this reason, it is important to correctly assess their antibiotic resistance before suggesting their technological application [48,49].

### 4.3. Adjunct Culture Implementation

Based on their metabolic features, few of the screened strains proved to be good candidates to be used as adjuncts for the production of raw milk hard cheese.

Strains 5959_Lbparacasei, 5998_Lbparacasei and 5296_Lbparacasei showed a good resistance to salt and lysozyme, in addition to a good recovery from the heat stress; furthermore, they did not show an excessively short Lag, but at the same time a good acidification capacity and rate. On the other hand, 5293_Lbparacasei, 5959_Lbparacasei and 5649_Lbparacasei, but also 5780_Lbparacasei and 5296_Lbparacasei to a lesser extent, produced more ketone compounds that could better improve the volatile profile of dairy products.

Thus, the two best strains should be 5959_Lbparacasei and 5296_Lbparacasei, provided that resistance to fusidic acid for the first and to vancomycin for the latter are correctly measured before their employment. Perspectives for future studies are the application of the isolated strains in cheesemaking trials in order to test their effectiveness as adjunct culture in influencing the cheese aroma and monitor the absence of interference with the starter LAB acidification activity [50]. Especially for cheese varieties that use artisanal undefined cultures prepared by back-slopping procedure, the addition of autochthonous adjunct cultures is suggested to reduce batch-to-batch variability among productions [51].

This work offers a focus on the adaptive response to the cheesemaking stresses of NSLAB strains known to play a fundamental role in the aroma formation of the important category of raw milk hard cheeses ripened for long periods. This knowledge is useful not only to better understand the dynamics behind the evolution of the complex raw milk microbiota through cheese manufacturing until the cheese ripens, but also to formulate adjunct cultures specifics for this cheese category.

It is worth specifying that for many traditional PDO cheeses, the use of adjunct cultures is not allowed by the PDO regulation. However, in some cases, the supplementation of specific autochthonous strains during cheesemaking is permitted to enrich the acidifying performance of the natural whey culture, such as in the case of Grana Padano PDO [52]. Autochthonous strains have the particularity to be characteristic biotypes of raw milk or natural whey starter that are able to adapt to and are selected by the cheesemaking parameters, meaning they are strains characteristic of that matrix in those technological conditions. Autochthonous strains could be also used to implement adjunct cultures to specifically improve the aroma of PDO cheeses without significantly changing the other features. This could represent a tool to cope with the broad issue of the production of cheese with less complex flavor, a phenomenon that seems to be correlated with biodiversity diminishing along the dairy chain and possibly resulting as a consequence of the improvement in the hygiene of milk and the need for standardization of ripening [53]. Microbial diversity is in fact considered a key factor in the quality of traditional cheeses, and cases of biodiversity loss in LAB have been already reported for Grana Padano PDO (Trentingrana) cheese by Morandi et al. (2019) [54]. The authors monitored over two years the microbial characteristics of natural whey culture used as a primary starter and found a correlation between microbial diversity reduction with changes in the cheese’s volatile organic compounds profile, concluding that the recognition of the microbial depletion drivers should be necessary to preserve the cheese quality and prevent the loss of typical cheese traits.

However, when choosing a selected- or mixed-strains adjunct cultures to boost the cheese flavor, the cheese maker should be aware that this may not be the most suitable solution to ensure broad species diversity [14], and that the cultures must be purchased from private companies since their production requires facilities and know-how generally not present in traditional cheese factories. An alternative could be the optimization of a natural adjunct culture rich in NSLAB from the raw milk microbiota [55], followed by its management using a back-slopping system.

Finally, the results obtained in this study can also be useful to select strains suitable for alternative applications. Strains with interesting aromatic capabilities, such as 5293_Lbparacasei, 5649_Lbparacasei and 5780_Lbparacasei, could be good candidates as adjunct strains for uncooked cheese production, according to their heat sensitivity. Other examples are the isolates 5993_Lbparacasei, which was able to grow very well using D-fructose and could be used as a starter in the fermentation of plant-based food matrices for the production of cheese analogues [56,57], or 5293_Lbparacasei, able to metabolize D-Salicin and D-Cellobiose and thus potentially applicable in the fermentation of food by-products [58].

## Figures and Tables

**Table 1:** Isolates and reference strains selected for phenotypic studies: viable cells concentration (log CFU/mL) after their growth in MRS broth and UHT partially skimmed milk (UPSM) for 20 h at 37 °C (anaerobic condition).

Strain Id	MRS Broth	UPSM
2094_Lbcasei_C	9.15	9.77
4186_Lbparacasei_C	9.23	8.19
5293_Lbparacasei	8.62	7.96
5296_Lbparacasei	8.91	8.78
5569_Lbparacasei	8.96	8.55
5649_Lbparacasei	9.28	8.93
5780_Lbparacasei	9.38	8.81
5785_Lbparacasei	9.03	8.52
5959_Lbparacasei	9.09	8.46
5963_Lbparacasei	9.37	8.83
5993_Lbparacasei	9.22	7.98
5998_Lbparacasei	8.12	8.64

**Table 2:** Data calculation to obtain values for statistical analysis and metabolic functional diversity indices [29].

Estimation	Formula	C Source Utilization	Chemical Sensitivity
Average Well Color Development (AWCD)	$AWCD=\sum_{i=1}^{n}\frac{\left(ODi-R\right)}{n}$	*ODi* = raw OD value of each well from 1 to 9 column *R* = OD value of negative control well *n* = number of substrates analyzed (71)	*ODi* = raw OD value of each well from 10 to 12 columns *R* = half of the OD value of positive control well *n* = number of substrates analyzed (23)
Absorbance Ratios	$R_{si}=\frac{\left({OD}_{i}-R\right)}{AWCD}$		

**Table 3:** Features of raw milk samples after the spontaneous fermentation (incubation for 60 d at 8 °C anaerobically). Intensity scale of odor descriptors: 0 = absent, 5 = strong.

Grana Padano PDO Dairy	Province of Origin	Lactobacilli (log CFU/mL)	pH	Initial Isolates (n) (tot. = 122)	Strains after Morphological Screening (n) (tot. = 68)	Strains Belonging to *Lacticaseibacillus* spp. (n) (tot. = 31)	Strains after AFLP Screening (n) (tot. = 10)	Odor Evaluation			
								Expired Milk	Acidic (Yogurt-like)	Cheese	Notes
D1	Bergamo	7.39	4.40	7	4	3	1	0	4	3	High gas formation, fizzy
D2	Brescia	5.90	4.60	6	3	1	1	0	4	2	Slight fruity notes
D3	Brescia	7.08	4.52	8	3	2	-	2	2	3	-
D4	Brescia	7.58	4.59	4	3	1	1	0	2	5	-
D5	Cremona	6.34	4.53	5	3	2	1	2	2	2	-
D6	Cremona	7.67	4.60	6	4	1	-	0	2	5	-
D7	Cuneo	6.62	4.50	6	3	2	-	0	2	3	-
D8	Lodi	7.70	4.50	6	4	2	-	0	2	5	-
D9	Lodi	7.95	4.69	8	5	2	1	0	3	2	High gas formation, fizzy
D10	Mantova	7.75	4.60	5	4	1	-	0	2	3	-
D11	Mantova	7.59	4.50	8	3	2	-	0	0	5	-
D12	Padova	6.94	4.40	7	4	2	1	0	4	2	High gas formation, fizzy
D13	Pavia	6.82	4.50	4	3	-	-	0	5	1	Slight fruity notes
D14	Piacenza	7.39	4.50	7	3	2	1	0	2	5	-
D15	Piacenza	5.76	4.56	7	3	0	-	0	2	4	-
D16	Trento	7.95	4.45	5	3	1	1	0	3	5	-
D17	Verona	6.53	4.50	5	3	1	1	0	3	3	-
D18	Verona	7.53	4.50	5	4	3	-	0	3	4	-
D19	Vicenza	8.55	4.59	6	3	1	-	0	4	5	-
D20	Vicenza	7.46	4.31	7	3	2	1	0	1	5	-

**Table 4:** Volatile compounds produced by the isolated strains after their anaerobic incubation in UHT partially skimmed milk for 4 days at 37 °C. Data (mg/L) are expressed as mean ± standard deviation (*n* = 2). Kovats indexes are reported in Table S1.

Compound Class	Aromatic Compound	Odor Type ^(a)^	Strain ID ^(b)^											
			2094_C	4186_C	5293	5296	5569	5649	5780	5785	5959	5963	5993	5998
**Acids**	Acetic acid	sharp pungent sour vinegar	0.215 ± 0	n.d.	n.d.	n.d.	n.d.	n.d.	n.d.	n.d.	n.d.	n.d.	n.d.	n.d.
	Hexanoic acid	sour fatty sweet cheese	0.049 0.034	0.003 ± 0	0.007 ± 0.001	n.d.	n.d.	n.d.	n.d.	n.d.	n.d.	n.d.	n.d.	n.d.
	Decanoic acid	sour, creamy, buttery, fatty	n.d.	0.005 ± 0	n.d.	0.002 ± 0.001	0.003 ± 0	n.d.	n.d.	0.011 ± 0.005	0.004 ± 0.001	0.004 ± 0.001	0.003 ± 0	0.003 ± 0.001
	Octanoic acid	fatty waxy rancid oily vegetable cheesy	0.021 ± 0.014	0.007 ± 0	0.005 ± 0	0.005 ± 0.002	0.006 ± 0.001	n.d.	0.026 ± 0.005	0.017 ± 0.007	0.007 ± 0.002	0.007 ± 0.001	0.006 ± 0	0.006 ± 0.002
**Acids sum**			0.285	0.015	0.012	0.007	0.009	n.d.	0.026	0.028	0.011	0.011	0.009	0.009
**Alcohols**	2-Furanmethanol	alcoholic chemical musty sweet caramel bread coffee	n.d.	0.004 ± 0.001	n.d.	n.d.	n.d.	n.d.	n.d.	n.d.	n.d.	n.d.	n.d.	n.d.
	Ethanol	strong alcoholic ethereal medical	0.003 ± 0.002	0.002 ± n.d.	0.001 ± 0	n.d.	0.001 ± 0	0.006 ± 0.001	0.01 ± 0.001	0.003 ± 0	0.001 ± 0	0.001 ± 0.001	0.001 ± 0	n.d.
**Alcohols sum**			0.003	0.006	0.001	n.d.	0.001	0.006	0.01	0.003	0.001	0.001	0.001	n.d.
**Ketons**	2,3-Butanedione	sweet, creamy, buttery, pungent caramellic nuance	n.d.	n.d.	0.001 ± 0	0.001 ± 0	n.d.	0.002 ± 0	n.d.	n.d.	n.d.	n.d.	n.d.	n.d.
**Ketons**	2-Heptanone	cheese, fruity, ketonic, green banana, with a creamy nuance	n.d.	0.002 ± 0	n.d.	n.d.	n.d.	n.d.	n.d.	n.d.	n.d.	n.d.	n.d.	n.d.
	Acetoin	sweet buttery creamy dairy milky fatty	0.114 ± 0.073	0.015 ± 0.001	n.d.	n.d.	n.d.	0.037 ± 0.003	n.d.	n.d.	0.014 ± 0	n.d.	n.d.	n.d.
	Acetone	solvent ethereal apple pear	n.d.	n.d.	n.d.	n.d.	n.d.	0.011 ± 0.002	0.001 ± 0	n.d.	n.d.	n.d.	n.d.	n.d.
	Dimethyl sulfone	sulfurous burnt	0.011 ± 0.01	0.001 ± 0	0.001 ± 0	n.d.	n.d.	n.d.	n.d.	n.d.	n.d.	n.d.	n.d.	n.d.
**Ketons sum**			0.125	0.019	0.003	0.001	n.d.	0.049	0.001	n.d.	0.014	n.d.	n.d.	n.d.
**Others**	2,4-Dimethyl-1-heptene	absent	n.d.	0.005 ± 0	n.d.	n.d.	n.d.	n.d.	n.d.	n.d.	n.d.	n.d.	n.d.	0.002 ± 0.001
	3-Methyl-3-buten-1-ol	sweet fruity	n.d.	n.d.	n.d.	n.d.	0.001 ± 0	n.d.	n.d.	n.d.	n.d.	n.d.	n.d.	n.d.
	alpha-Pinene	Woody, piney, and turpentine-like, a fresh herbal lift	n.d.	n.d.	0.001 ± 0	0.001 ± 0	0.001 ± 0	n.d.	n.d.	0.004 ± 0.001	0.002 ± 0.001	0.003 ± 0.001	n.d.	0.003 ± 0.001
**Others sum**			n.d.	0.005	0.001	0.001	0.002	n.d.	n.d.	0.004	0.002	0.003	n.d.	0.005

^(a)^ Odor types were retrieved from TGSC (www.thegoodscentscompany.com/allodor.html, accessed on 23 March 2023). ^(b)^ See Figure 1 for strains’ species. _C = reference strains used as control. n.d. = not detected.

## Data Availability

The data used to support the findings of this study can be made available by the corresponding author upon request.

## Supplementary Materials

The following supporting information can be downloaded at: https://www.mdpi.com/article/10.3390/foods12213949/s1. Table S1: identification of the compound produced by the strains as measured using HS-SPME GC-MS. Reference Kovats index literature: [59,60,61,62,63,64,65,66,67,68,69,70,71]. Figure S1: discrimination between the *Lacticaseibacillus casei/paracasei* and *Lacticaseibacillus rhamnosus* species of the isolates according to their melting temperature curve analysis. The reference strains are indicated with their numerical ID in red scale lines; reference strains are in green (*Lacticaseibacillus paracasei*) and blue (*Lacticaseibacillus rhamnosus*) lines. Figure S2: ten most contributing (%) variables in defining the strains’ differentiation on the first (**A**) and the second (**B**) principal component. See Figure 8 in the article for the principal component analysis plot.
