# Supplementary material for: Lacticaseibacillus Strains Isolated from Raw Milk: Screening Strategy for Their Qualification as Adjunct Culture in Cheesemaking
Source: Foods. 2023 Oct 29;12(21):3949. doi: 10.3390/foods12213949 (PMC10648420; doi:10.3390/foods12213949)
Supplement: Supplementary file 1 [file foods-12-03949-s001.zip › foods-2659240-supplementary.pdf]

Supplementary material

Table S1. Identification of the compound produced by the strains and measured by means of HS-SPME GC-MS. Reference Kovats index literature: [55–67]

Compound	CAS number	Retention time (min)	Kovats index (calculated)	Kovats index (literature)(a)	Reference Kovats index literature(a)
Acetone	67-64-1	1.53	864.47	847	[59]
2,4-Dimethyl-1-heptene	19549-87-2	1.72	889.47	885	[60]
Ethanol	64-17-5	2.05	932.89	931	[61]
2,3-Butanedione	431-03-8	2.41	980.26	980	[62]
α-Pinene	80-56-8	2.76	1011.43	1011	[63]
2-Heptanone	110-43-0	5.85	1167.25	1169	[64]
3-Methyl-3-buten-1-ol	763-32-6	7.57	1238.19	1237	[65]
Acetoin	513-86-0	8.56	1277.17	1277	[66]
Acetic acid	64-19-7	14.7	1521.01	1498	[67]
2-Furanmethanol	98-00-0	17.86	1655.9	1656	[68]
Dimethyl sulfone	67-71-0	21.83	1904.26	1906	[69]
Hexanoic acid	142-62-1	22.01	1923.4	1889	[70]
Octanoic acid	124-07-2	23.82	2115.96	2106	[71]
Decanoic acid	334-48-5	25.23	2265.96	2266	[71]

(a) Retrieved from the NIST database (www.nist.gov/)

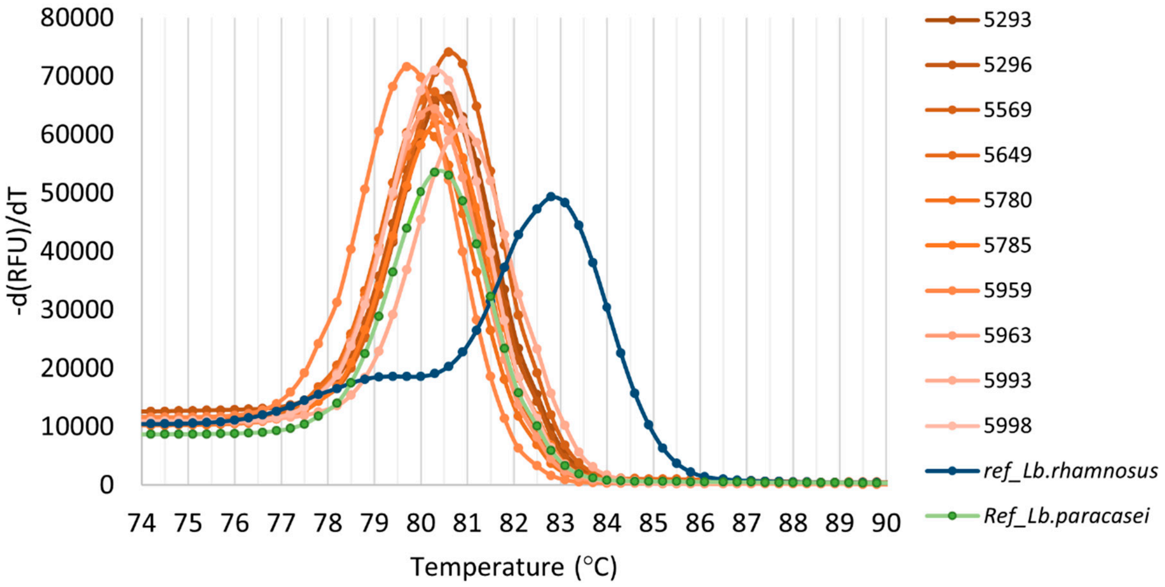

Figure S1. Discrimination among the *Lacticaseibacillus casei/paracasei* and *Lacticaseibacillus rhamnosus* species of the isolates according to the melting temperature curve analysis. The reference strains are indicated with their numerical ID in red scale lines; references strains are in green (*Lacticaseibacillus paracasei*) and blue (*Lacticaseibacillus rhamnosus*) line.

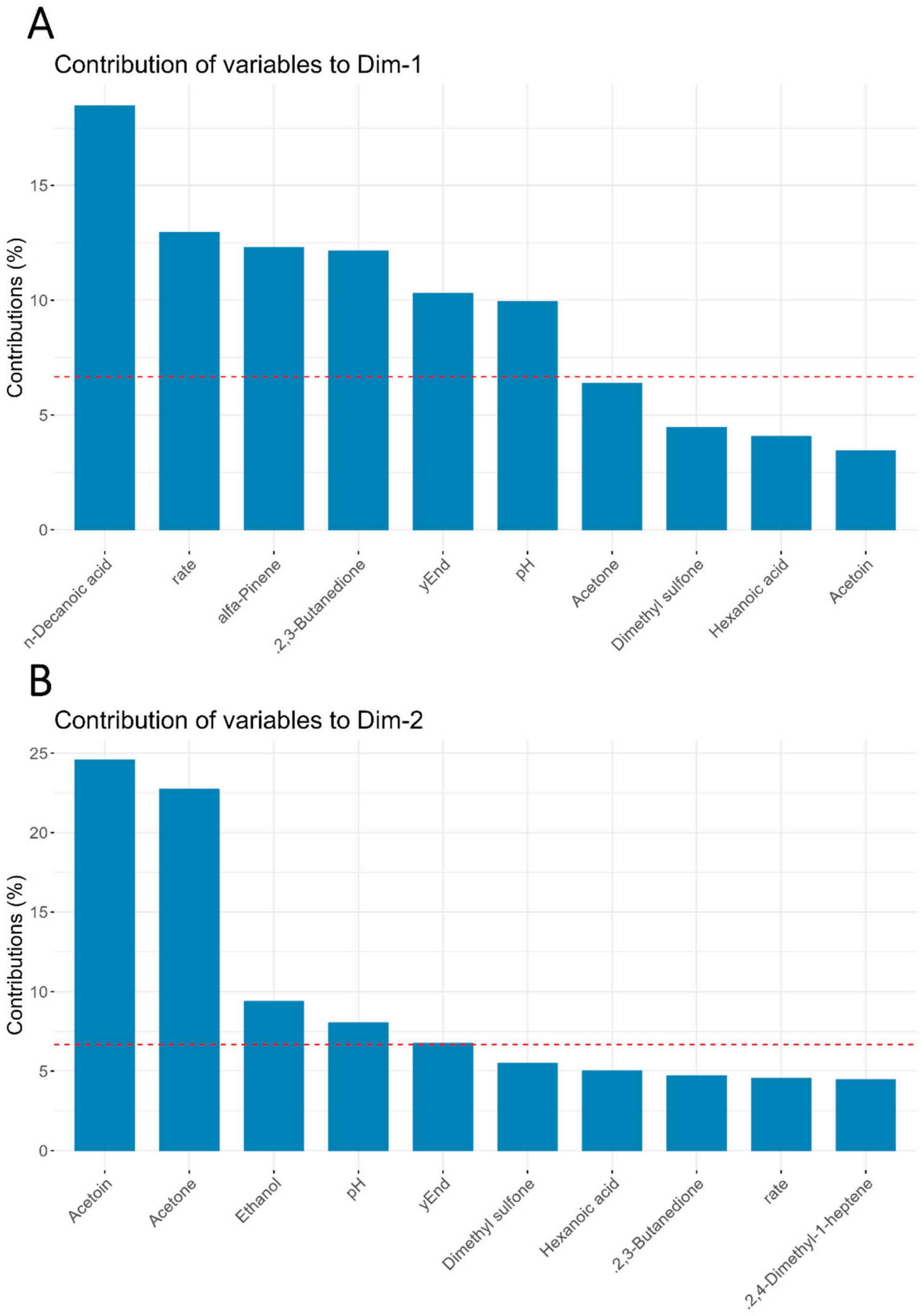

Figure S2. Ten most contributing (%) variables in defining the strains' differentiation on the first (A) and the second (B) principal component. See **Figure 8** in the article for the principal component analysis plot.

References

59. Waggott, A.; Davies, I.W. Identification of Organic Pollutants Using Linear Temperature Programmed Retention Indices (LTPRIs) 1984.
60. Muresan, S.; Eillebrecht, M.A.J.L.; Rijk, T.C. de; Jonge, H.G. de; Leguijt, T.; Nijhuis, H.H. Aroma Profile Development of Intermediate Chocolate ProductsI. Volatile Constituents of Block-Milk. Food Chemistry 2000, 68, 167–174, doi:10.1016/S0308-8146(99)00171-5.
61. Bonastre, J.; Grenier, P. Contribution à l'étude de La Polarité Des Phases Stationnaires En Chromatographie Gaz-Liquide. III. Calcul Des Coefficients d'activité Relatifs et Des Indices de

Rétention de Quelques Alcools Aliphatiques. Bulletin de la Société Chimique de France 1968, 1, 118–125.
62. Umano, K.; Shoji, A.; Hagi, Y.; Shibamoto, T. Volatile Constituents of Peel of Quince Fruit, Cydonia Oblonga Miller. J. Agric. Food Chem. 1986, 34, 593–596, doi:10.1021/jf00070a003.
63. Umano, K.; Shibamoto, T. A New Method of Headspace Sampling: Grapefruit Volatiles. In Proceedings of the Proceedings of the 10th International Congress of Essential Oils, Fragrances and Flavors; Elsevier Ltd: New York, 1988; pp. 981–998.
64. Umano, K.; Hagi, Y.; Shibamoto, T. Volatile Chemicals Identified in Extracts from Newly Hybrid Citrus, Dekopon (Shiranuhi Mandarin Suppl. J.). J. Agric. Food Chem. 2002, 50, 5355–5359, doi:10.1021/jf0203951.
65. Yuhong, T. Research Advances on the Essential Oils from Leaves of Eucalyptus. Food and Fermentation Industries 2007, 33, 139.
66. Kim, T.H.; Kim, T.H.; Shin, J.H.; Yu, E.J.; Kim, Y.-S.; Lee, H.J. Characteristics of Aroma-Active Compounds in the Pectin-Elicited Suspension Culture of Zanthoxylum Piperitum (Prickly Ash). Biotechnology Letters 2002, 24, 551–556, doi:10.1023/A:1014812508441.
67. Soria, A.C.; Martínez-Castro, I.; Sanz, J. Some Aspects of Dynamic Headspace Analysis of Volatile Components in Honey. Food Research International 2008, 41, 838–848, doi:10.1016/j.foodres.2008.07.010.
68. Kim, J.H.; Ahn, H.J.; Yook, H.S.; Kim, K.S.; Rhee, M.S.; Ryu, G.H.; Byun, M.W. Color, Flavor, and Sensory Characteristics of Gamma-Irradiated Salted and Fermented Anchovy Sauce. Radiation Physics and Chemistry 2004, 69, 179–187, doi:10.1016/S0969-806X(03)00400-6.
69. Chung, H.Y.; Yung, I.K.S.; Ma, W.C.J.; Kim, J.-S. Analysis of Volatile Components in Frozen and Dried Scallops (Patinopecten Yessoensis) by Gas Chromatography/Mass Spectrometry. Food Research International 2002, 35, 43–53, doi:10.1016/S0963-9969(01)00107-7.
70. Peng, C.T. Prediction of Retention Indices: V. Influence of Electronic Effects and Column Polarity on Retention Index. Journal of Chromatography A 2000, 903, 117–143, doi:10.1016/S0021-9673(00)00901-8.
71. Welke, J.E.; Manfroi, V.; Zanus, M.; Lazarotto, M.; Alcaraz Zini, C. Characterization of the Volatile Profile of Brazilian Merlot Wines through Comprehensive Two Dimensional Gas Chromatography Time-of-Flight Mass Spectrometric Detection. Journal of Chromatography A 2012, 1226, 124–139, doi:10.1016/j.chroma.2012.01.002.
